# Cigarette smoking, nicotine dependence and anxiety disorders: a systematic review of population-based, epidemiological studies

**DOI:** 10.1186/1741-7015-10-123

**Published:** 2012-10-19

**Authors:** Steven Moylan, Felice N Jacka, Julie A Pasco, Michael Berk

**Affiliations:** 1 Deakin University School of Medicine, Barwon Health, Geelong, Victoria, Australia; 2 Department of Psychiatry, Melbourne University, Parkville, Victoria, Australia; 3 NorthWest Academic Centre, Department of Medicine, The University of Melbourne, St Albans, Victoria, Australia; 4 Orygen Youth Health Research Centre, Centre for Youth Mental Health, Parkville, Victoria, Australia; 5 The Florey Institute for Neuroscience and Mental Health, Parkville, Victoria, Australia

**Keywords:** agoraphobia, anxiety disorder, cigarette smoking, epidemiology, generalized anxiety disorder, nicotine dependence, obsessive-compulsive disorder, panic disorder, post-traumatic stress disorder, specific phobia

## Abstract

**Background:**

Multiple studies have demonstrated that rates of smoking and nicotine dependence are increased in individuals with anxiety disorders. However, significant variability exists in the epidemiological literature exploring this relationship, including study design (cross-sectional versus prospective), the population assessed (random sample versus clinical population) and diagnostic instrument utilized.

**Methods:**

We undertook a systematic review of population-based observational studies that utilized recognized structured clinical diagnostic criteria (*Diagnostic and Statistical Manual of Mental Disorders *(DSM) or *International Classification of Diseases *(ICD)) for anxiety disorder diagnosis to investigate the relationship between cigarette smoking, nicotine dependence and anxiety disorders.

**Results:**

In total, 47 studies met the predefined inclusion criteria, with 12 studies providing prospective information and 5 studies providing quasiprospective information. The available evidence suggests that some baseline anxiety disorders are a risk factor for initiation of smoking and nicotine dependence, although the evidence is heterogeneous and many studies did not control for the effect of comorbid substance use disorders. The identified evidence however appeared to more consistently support cigarette smoking and nicotine dependence as being a risk factor for development of some anxiety disorders (for example, panic disorder, generalized anxiety disorder), although these findings were not replicated in all studies. A number of inconsistencies in the literature were identified.

**Conclusions:**

Although many studies have demonstrated increased rates of smoking and nicotine dependence in individuals with anxiety disorders, there is a limited and heterogeneous literature that has prospectively examined this relationship in population studies using validated diagnostic criteria. The most consistent evidence supports smoking and nicotine dependence as increasing the risk of panic disorder and generalized anxiety disorder. The literature assessing anxiety disorders increasing smoking and nicotine dependence is inconsistent. Potential issues with the current literature are discussed and directions for future research are suggested.

## Background

Anxiety disorders (ADs) represent the most common mental illness diagnoses across many countries [1,2] and are associated with significant impairment to health and quality of life [3,4]. Multiple population-based epidemiological studies have identified increased rates of smoking amongst individuals with mental illness, and increased rates of mental illness amongst smokers [5]. Specifically, ADs have been reported to be associated with increased rates of smoking, increased consumption of cigarettes per smoker, and lower rates of smoking cessation than non-anxiety disordered control groups [6,7]. In addition, increased anxiety symptoms as measured on symptom scales appear to be correlated with increased rates of smoking [8].

The relationship between ADs, smoking behavior and nicotine dependence (ND) could be explained by three non-mutually exclusive relationships. Firstly, smoking behavior and/or ND may increase the chances of developing an AD. Potential mechanisms underpinning this include adverse effects of smoking on neurodevelopment and neurotransmitter pathways modulating anxiety that may predispose individuals to developing enhanced anxiety, or direct effects to the respiratory and autonomic systems that may alter physical responses to anxiety provoking situations [9,10]. Secondly, ADs may increase smoking behavior and the risk for ND. Mechanisms for this could include a propensity for those with increased anxiety to commence smoking, or use of cigarettes as an anxiolytic self-treatment [11,12]. Thirdly, the relationship may be underpinned by a shared vulnerability factor or group of factors that increase the likelihood of smoking, ND and AD development (for example, low socioeconomic status) [2,13].

However, a number of inconsistencies exist within the current literature examining the relationships between cigarette smoking, ND and ADs. Firstly, many currently available population-based epidemiological investigations have utilized clinical symptom severity scales rather than validated diagnostic instruments to make AD diagnoses. Secondly, many studies utilizing validated diagnostic instruments have been cross-sectional, and therefore unable to provide insight into the direction of causality underpinning the association. Thirdly, many investigations have been drawn from clinical populations as opposed to whole population samples, potentially introducing selection biases. An accurate assessment of the association between cigarette smoking, ND and validated ADs, including direction of causality and potential variation amongst differing anxiety disorder subgroups or populations, may help inform targeted interventions in at risk populations.

This paper aims to critically review the available epidemiological studies that have utilized general population samples and validated diagnostic instruments based upon recognized *Diagnostic and Statistical Manual *(DSM) [14] or *International Classification of Diseases *(ICD) systems [15], exploring the relationship between cigarette smoking, ND and ADs. Structured diagnostic instruments have advantages, as they are replicable across studies, and in some cases include an interview (for example, the Composite International Diagnostic Interview (CIDI)). These interviews often document date of onset and recent symptom expression, allowing for assessments of temporality. A particular focus for this review is on studies that have utilized prospective designs and can therefore provide insight into the direction of the potential underlying causal relationships.

## Methods

### Systematic review search strategy

A systematic review of the English language literature exploring relationships between ADs, ND and cigarette smoking was undertaken. The aim was to provide a descriptive overview of the available literature, with a focus on information that could inform understanding of direction of causality. A computerized search strategy of medical databases PubMed and EMBASE utilized the following search strategies, was limited to English language literature and human studies. No date restrictions were placed. PubMed: 'Anxiety Disorders' (MeSH) AND (Smoking(Title/Abstract) OR Tobacco(Title/Abstract) OR Nicotine(Title/Abstract) OR Cigarette(Title/Abstract)) AND Anxiety AND ('humans' (MeSH terms) AND English); EMBASE: '#1 AND #2 AND #3' where #1 = ''anxiety disorder'/exp AND (humans)/lim AND (english)/lim AND (embase)/lim', #2 = 'smoking:ab OR tobacco:ab OR cigarette:ab OR nicotine:ab AND (humans)/lim AND (english)/lim AND (embase)/lim' and #3 = ''anxiety'/exp AND (humans)/lim AND (english)/lim AND (embase)/lim'.

### Extraction of references

All extracted references were combined and duplicate references deleted. Titles and abstracts of all references were initially assessed for relevance to the review topic. Full texts of references identified as potentially containing relevant information were assessed against the predetermined inclusion criteria for quality and relevance. For completeness, bibliographies of extracted references were manually searched for further relevant references. Where relevant references were discovered, their bibliographies were manually searched.

### Inclusion criteria

The following inclusion criteria were applied to the available references. Included studies needed to utilize a random sample, drawn from the general population, investigating the association between ADs, ND and cigarette smoking. The included population must have been assessed for panic disorder (PD), generalized anxiety disorder (GAD), obsessive-compulsive disorder (OCD), social phobia (SP), specific phobia (SPP), post-traumatic-stress disorder (PTSD) and/or agoraphobia (AG), utilizing a recognized and validated structured diagnostic tool or documented clinician diagnosis in line with either ICD-9/ICD-10 or DSM-III or DSM-IV criteria. The criteria establishing cigarette smoking must have been clearly documented, either as dichotomous (yes/no) or through frequency of cigarette consumption. ND must have been diagnosed through a recognized diagnostic tool linked to ICD or DSM criteria. The criteria for ND mirror those of other substance dependence disorders, and include (amongst others) symptoms of tolerance, withdrawal, increasing use and difficulty quitting. All data must have represented new information and not replication of previous study results.

### Literature search and application of inclusion criteria

**Figure 1:**
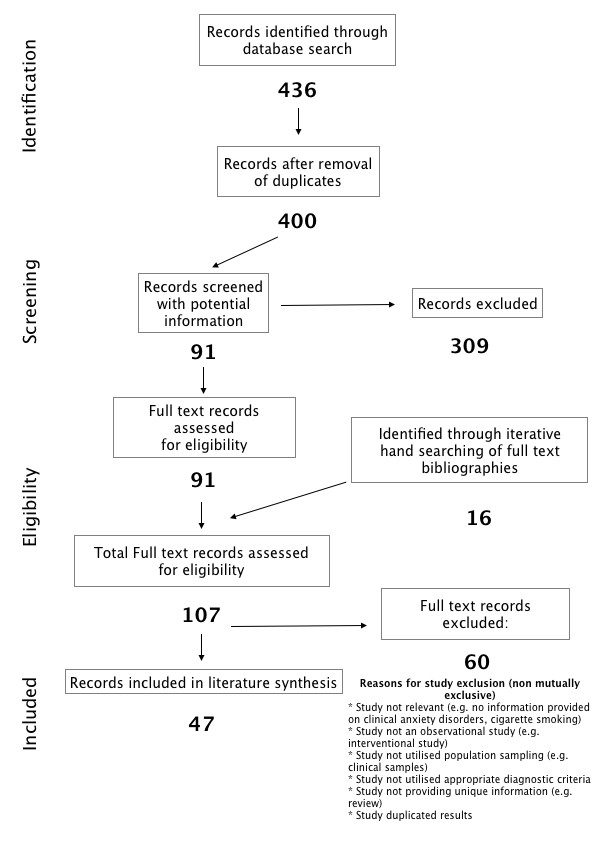
**Preferred Reporting Items for Systematic Reviews and Meta-Analyses (PRISMA) search strategy flow diagram**.

**Figure 2:**
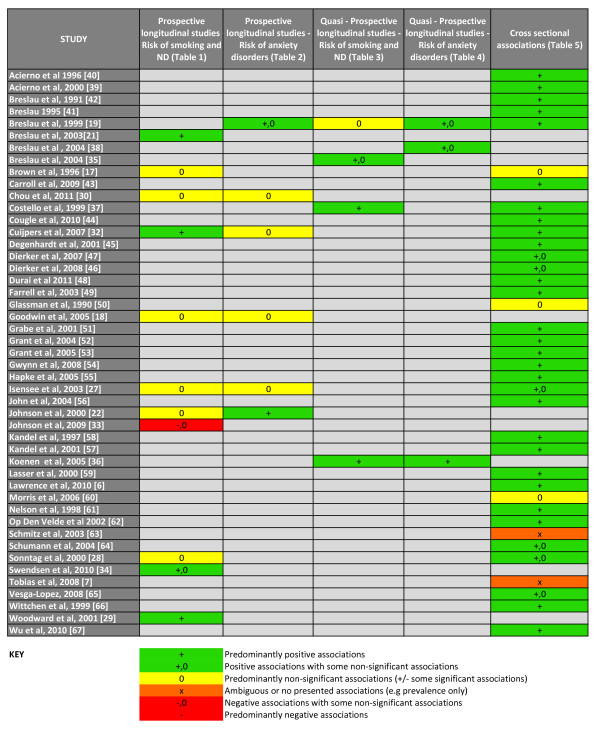
**Included studies: prospective, quasiprospective and cross-sectional association information obtained from each study**.

The structured computerized literature search was performed on 18 November 2011 yielded 298 references from PubMed and 138 references from EMBASE. In all, 36 records were duplicated after compilation of search results, leaving 400 unique records. Initial review of titles and abstracts revealed 91 records with potentially relevant information for the review. Manual searching of the bibliographies from these 91 references revealed 16 further potential references. Full text review of these 107 references revealed 47 studies meeting the inclusion criteria. See Figure 1 for search strategy flowchart and Figure 2 for breakdown of studies.

### Data extraction

All data were extracted upon the following variables: (1) study name, (2) study population, (3) study design (prospective, quasiprospective or cross-sectional), (4) definition of AD diagnosis, (5) definition of smoking status, (6) statistical parameters utilized and (7) primary results presented. A focus on measures of association (for example, odds ratio (OR), hazard ratio (HR)) was taken. Where available, both unadjusted and adjusted measures were extracted. Data were extracted into a preprepared structured Microsoft Excel database (Microsoft; Redmond, WA, USA).

## Results

There were 47 studies meeting inclusion criteria. The studies were broken down by type and direction of analysis (prospective, quasiprospective and cross-sectional) and are represented in Additional files 1, 2, 3, 4, 5.

### Prospective studies

A total of 13 studies were identified that utilized population-based samples to assess a prospective relationship between ADs, smoking behavior, and ND. These studies comprised random population samples drawn from the United States, New Zealand, Germany and The Netherlands, with some samples used in multiple studies. Discussion of the included studies has been grouped below based on the sample used.

### Influence of anxiety disorders on risk of later smoking or nicotine dependence

(A summary of this section is as follows (see also Additional file 1, Table S1): the best available evidence is equivocal, but suggests that certain baseline ADs are risk factors for onset of smoking and nicotine dependence, although results vary across studies and across different disorders. The best available studies failed to control for comorbid substance use disorders.)

The first published prospective data were drawn from the Oregon Adolescent Project Depression project, which randomly recruited adolescents (aged 14 years to 18 years) from high schools in Western Oregon, USA in 1987 to 1989 and followed them at two time points (1 year later, and on their 24th birthday). Assessment of ADs was undertaken utilizing the Schedule for Affective Disorders and Schizophrenia for School-Age Children (K-SADS) for DSM-III revision (DSM-III-R) [16], and smokers were defined as those smoking ≥ 3 times per week. Brown *et al. *[17] demonstrated no difference in the odds for incident smoking at 1-year follow-up in those with versus without any ADs at baseline, both unadjusted and after controlling for a variety of demographic and other risk factors. This finding of no association was replicated in the 24-year-old follow-up [18] although only PD, and not grouped ADs, was used as the exposure variable.

Breslau *et al. *[19] utilized a random population sample of 1,007 young adults (21 years to 30 years), drawn from the Detroit Epidemiologic Study, to examine PD as a predictor of cigarette smoking. Baseline assessment occurred in 1989 and three follow-ups (1990, 1992 and 1994) were conducted. Diagnosis of PD was made through use of the National Institute of Mental Health Diagnostic Interview Schedule [20] for DSM-III-R criteria, and 'daily smoking' was defined as smoking daily for ≥ 1 month. Risk of smoking onset was increased in those with PD at baseline (HR = 2.20 (95% CI 1.10 to 4.42)), but this significance was lost when controlled for the presence of major depressive disorder (MDD). Breslau *et al. *[21] extended their investigation, incorporating two further follow-ups (1999 and 2001) to assess the interaction between PTSD and ND over 10 years. After adjusting for gender, race and education, the odds of incident ND at follow-up was 4.03 (95% CI 2.10 to 7.72) in those with baseline PTSD versus those without a trauma history.

Johnson *et al. *[22] followed a random sample of adolescents from New York state, assessed at baseline in 1983 (mean age 14 years) and at two subsequent follow-ups in 1985 to 1986 (mean age 16 years) and 1991 to 1993 (mean age 22 years). Diagnosis of ADs (grouped and individual) at baseline was made using the Diagnostic Interview Schedule for Children (DIS-C) [23] to DSM-III criteria. Smoking assessment was by self-report, and categorized into smoking > 1 pack (20+ cigarettes) versus smoking < 1 pack (1 to 19 cigarettes) per day (not non-smokers). After adjusting for a variety of demographic and other risk factors no association was detected between adolescent anxiety disorder status and onset of smoking in adulthood.

The Early Developmental Stages of Psychopathology (EDSP) study has been utilized to assess the association between ADs, ND and smoking behavior. In this study, a random community cohort of 3,021 adolescents and young adults (age 14 years to 24 years) was sampled from metropolitan Munich. The cohort was assessed at baseline (1995) and two follow-ups, the first between 1996 and 1997 and the second between 1998 and 1999 (a third follow-up was subsequently conducted in 2005 to 2006) [24]. AD and ND diagnoses were made utilizing an updated version [25] of the Composite International Diagnostic Interview [26] for DSM-IV criteria and smoking was assessed by self-report. Isensee *et al. *[27] categorized participants from the EDSP into non-smokers, occasional smokers, non-dependent regular smokers and dependent regular smokers (see Additional file 1, Table S1 for definitions) and calculated odds ratios for incident smoking by baseline AD status. No associations were found between baseline AD status and odds of incident smoking. Sonntag *et al. *[28] extended this study in SP but once again found no association, although a positive association was found between those with social fears symptoms and later development of ND.

Woodward *et al. *[29] utilized a New Zealand birth cohort to compare the risk of DSM-IV ND between the ages of 18 and 21, dependent upon diagnosis of ADs between the ages of 14 to 16. A linear association was found between increasing number of ADs at age 14 to 16 (0 to 3+) and subsequent ND diagnosis, although this was not significant after controlling for childhood sexual abuse, alcohol abuse, parental changes and deviant peer affiliations.

Chou *et al. *[30] utilized the National Epidemiologic Survey on Alcohol and Related Conditions (NESARC) to investigate the association between ADs and ND in adults age 60 years and older (n = 8,012). Diagnoses were made utilizing the Alcohol Use Disorders and Associated Disabilities Interview Schedule for DSM-IV criteria (AUDADIS-IV) [31] and the risk of incident ND was assessed between baseline (2000 to 2001) and follow-up (2004 to 2005). No associations were found between baseline AD status and subsequent ND.

Cuijpers *et al. *[32] utilized data from the Netherlands Mental Health Survey and Incident Study (NEMESIS) to investigate the relationship between incident ADs (expressed as incident rate ratios) and previous smoking status. The NEMESIS study randomly recruited adults (18 years to 64 years) from 90 municipalities in The Netherlands, undertaking baseline assessments and 2 follow-ups at 1 year and 3 years (n = 4,796). The CIDI [26] was used for DSM-III-R diagnoses, and smoking was assessed by self-report, with participants placed into 4 categories (non-smokers, 1 to 9 cigarettes daily, 10 to 19 cigarettes daily and 20+ cigarettes daily). In a follow-up analysis, having 12-month (incidence rate ratio (IRR) 4.46 (*P *< 0.05)) and lifetime GAD (IRR 4.46 (*P *< 0.05)) was associated with increased risk of smoking onset at follow-up.

Johnson *et al. *[33] utilized the prospective follow-up of the full NESARC database (n = 34,653) to assess the impact of AD on smoking onset and persistence. Grouped ADs were associated with reduced daily smoking onset (OR 0.62 (95% CI 0.39 to 0.99)) when adjusting for demographics and socioeconomic status, but not with smoking persistence. Interestingly, comorbid substance use was an effect modifier; respondents with a comorbid substance use disorder (for example, alcohol, marijuana, amphetamines, opioids, sedatives, tranquilizers, cocaine, inhalants, hallucinogens, heroin, and other drugs) and AD demonstrated an increased risk of daily smoking onset (OR 2.22 (1.01 to 4.91)), whereas those without comorbid substance use disorder had a decreased risk of smoking onset (OR 0.43 (0.23 to 0.78)).

Most recently, Swendsen *et al. *[34] utilized a 10-year follow-up of 5,001 participants drawn from the National Co-morbidity Survey (NCS) to investigate mental disorders as a risk factor for onset of daily smoking or ND. The NCS, conducted in the US between 1990 and 1992, was a stratified multistage probability sample of 8,098 non-institutionalized residents (age 15 years to 54 years), utilizing the CIDI (V1.1), that assessed the interaction between smoking, ND and DSM-III-R mental disorders. The NCS2, performed in 2001 to 2002, was a 10-year re-interview of 5,001 participants from the NCS, but which utilized an updated version CIDI (V3.0) for DSM-IV criteria. After adjusting for sociodemographic characteristics, the odds of commencing daily smoking was increased for respondents with baseline PD, SP, GAD and specific phobia, but not for those with baseline PTSD or agoraphobia. In contrast, in those respondents with baseline daily smoking the odds of ND onset were raised in PTSD, agoraphobia and specific phobia, but not PD, SP or GAD. When considering the whole population odds of ND onset, having baseline PTSD, SP and specific phobia conferred an increased risk (see Additional file 1, Table S1).

### Smoking and nicotine dependence as risk factors for later anxiety disorders

(A summary of this section is as follows (see also Additional file 2, Table S2): the available prospective evidence associating smoking and nicotine dependence as risk factors for incident anxiety disorders is limited and heterogeneous. However, smoking has been demonstrated as a risk factor for grouped anxiety disorders, panic disorder and generalized anxiety disorder in a number of studies, although these findings are not replicated in all studies.)

Data from the Oregon Adolescent Depression Project were utilized to assess the relationship between baseline smoking status and incident ADs. Goodwin *et al. *[18] demonstrated an association between increased odds of PD diagnosis at age 24 in those with daily smoking at baseline versus those not smoking daily (OR 5.1 (2.4 to 10.5)), which remained significant after controlling for other ADs and parental risk factors. No other associations were found.

Utilizing data from the Detroit Epidemiologic Study, Breslau *et al. *[19] found increased risk of subsequent PD onset in individuals with prior daily smoking even when controlling for gender and MDD (HR 13.13 (4.41 to 39.10)). In addition, prior daily smokers who continued to smoke were more likely to experience incident PD (HR 14.46 (4.81 to 43.5)) when controlled for gender and MDD.

In the New York Adolescent Cohort, relationships were discovered between odds of adult ADs when grouped (OR 10.78 (1.48 to 78.55)), GAD (OR 5.53 (1.84 to 16.66) and PD (OR 15.58 (2.31 to 105.14) when comparing baseline > 1 pack per day smokers versus < 1 pack per day smokers [22]. Data from the EDSP studies [27] demonstrated relationships between increased incident PD, agoraphobia, SP and PTSD when comparing baseline ND smokers versus non-users, however all associations became non-significant when controlled for comorbid conditions at baseline (depressive disorders, panic attacks, other ADs, alcohol and drug disorders, and eating disorders). In the NESARC study, Chou *et al. *[30] assessed the relationship between ND at baseline and subsequent ADs, finding no associations.

Cuijpers *et al. *[32] utilized data from the NEMESIS to investigate the relationship between incident ADs (expressed as incident rate ratios) and past smoking status. Smoking at 1-year follow-up was associated with increased incidence of grouped ADs (IRR 1.77 (1.10 to 2.86)) and GAD (IRR 3.80 (1.09 to 13.21)) after controlling for demographics and other risk factors. No other relationships were found (see Additional file 2, Table S2).

### Quasiprospective studies

(A summary of this section is as follows: a small number of studies have utilized a single time point analysis and retrospective self-report patient data to draw quasiprospective associations between smoking, ND and ADs. These studies generally indicate increased smoking behaviors or nicotine dependence in individuals with pre-existing anxiety disorders, and vice versa, although studies are limited by the retrospective nature of data.)

### Baseline anxiety disorders and risk of smoking or nicotine dependence

See also Additional file 3, Table S3. Breslau *et al. *[35] examined the 4,411 participants who completed the tobacco supplement of the NCS to assess the interaction between smoking, ND and DSM-III-R ADs, utilizing discrete time survival models with ADs as time dependent variables and controlling for race, gender, education and age. The onset of daily smoking was the age at which respondents first smoked daily for ≥ 1 month. Increased odds for daily smoking were found in patients with pre-existing (OR 1.9 (1.05 to 3.7)) or currently active GAD (OR 2.1 (1.1 to 3.9)), pre-existing (OR 1.6 (1.3 to 1.8)) or currently active specific phobia (OR 1.5 (1.3 to 1.8)), pre-existing (OR 1.5 (1.2 to 1.7)) or currently active SP (OR 1.3 (1.1 to 1.6)), and pre-existing (OR 2.1 (1.6 to 2.9)) or currently active PTSD (OR 2.0 (1.4 to 2.9)).

Breslau *et al. *[35] further investigated the odds of smoking persistence and the transitioning from daily smoking to ND based upon pre-existing and currently active ADs. Increased odds of transitioning from daily smoking to ND were found in individuals with pre-existing and currently active agoraphobia, specific phobia, SP and PTSD. Interestingly, having pre-existing, but not currently active, PD was also strongly associated with increased odds of daily smoking to ND transition (OR 5.8 (3.0 to 11.6)). No associations existed between odds of smoking persistence and any pre-existing AD.

Koenen *et al. *[36] utilized data from the national Vietnam Era Twin (VET) registry and retrospective self-report of age of onset to test prospective onset associations between DSM-III-R ADs and ND. Associations were controlled for various demographic and other risk factors and time-dependent covariates (conduct disorder, MDD, alcohol and drug abuse or dependence) were entered into models. The results demonstrated that pre-existing PTSD was associated with increased odds of subsequent ND (OR 1.73 (1.38 to 2.17)). Utilizing the Greater Smoky Mountain Study (GSMS), a longitudinal representative study of 4,500 children (aged 9 years, 11 years and 13 years) from western North Carolina, USA, Costello *et al. *[37] found children, both boys and girls, with any AD were more likely to commence smoking than those without an AD (see Additional file 3, Table S3).

#### Smoking and nicotine dependence and risk of incident anxiety disorders

See also Additional file 4, Table S4. Breslau *et al. *[38] utilized the NCS, including respondent recall about their age of smoking and ND onset, to assess the effect of these parameters on developing ADs. Adjusting for demographic characteristics, pre-existing daily smoking (defined as onset > 1 year prior to disorder onset) was associated with increased odds of PD (OR 2.6 (1.2 to 5.4)) and agoraphobia (OR 4.4 (2.3 to 8.2)). The role of ND was assessed across all ADs. In this analysis, ND smokers and non-ND smokers maintained increased odds of PD and agoraphobia, but no other ADs. The only other associations were found in relation to past smokers (without ND) who exhibited decreased odds of PTSD (OR 0.2 (0.1 to 0.5)) when controlled for demographics and other pre-existing psychiatric disorders. Breslau *et al. *[38] extended their study by comparing the age of smoking onset (early vs not early; see Additional file 4, Table S4 for definitions), standardized pack years of smoking and time since quitting against odds of AD diagnosis. No association was found between early onset smoking and ADs, but increased years since quitting was associated with decreased odds of subsequent PD (OR 0.5 (0.4 to 0.7)), agoraphobia (OR 0.5 (0.5 to 0.8)) and SP (OR 0.6 (0.4 to 0.8)). The associations between standardized pack years of smoking were not significant in all ADs except PD, where increased pack years of smoking appeared protective in current smokers but a risk factor in past smokers, and GAD where increased pack years was associated with increased odds of GAD in both current and past smokers.

In a separate analysis utilizing a subsample of NCS data, Breslau *et al. *[19] investigated the interaction between smoking characteristics and subsequent onset of PD. Significant relationships were discovered between prior daily smoking (HR 2.93 (1.84 to 4.66)) and smoking persistence in prior daily smokers (HR 3.18 (1.99 to 5.10)) and subsequent onset of PD. In addition, pre-existing ND was associated with increased odds of subsequent PTSD onset (OR 2.24 (1.78 to 2.83)) in the aforementioned study drawn from the VET registry [36].

### Cross-sectional studies

(A summary of this section is as follows (see also Additional file 5, Table S5): a large number of studies have reported cross-sectional relationships between cigarette smoking, nicotine dependence and anxiety disorders. Many demonstrate higher rates of smoking and nicotine dependence in those with anxiety disorders, and vice versa. However, their utility is limited due to their inherent inability to provide insight into direction of causality.)

Almost all studies included in this review reported cross-sectional associations between smoking and/or ND and ADs. Studies providing cross-sectional information [6,7,17,19,23,27,28,32,39-67] are listed in Additional file 5, Table S5. Descriptions of some selected larger studies utilizing population-based data are detailed below.

### Smoking or nicotine dependence by anxiety disorder status

Lasser *et al. *[59] utilized the NCS to demonstrate increased rates of current and lifetime smoking in respondents with current and lifetime SP (39.5% and 54%), agoraphobia (38.4% and 58.9%), PD (35.9% and 61.3%), specific phobia (40.3% and 57.8%), PTSD (45.3% and 63.3%) and GAD (46% and 68.4%) when controlling for gender, age and geographical region. Utilizing the NCS-R data, Cougle *et al. *[44] explored the role of comorbidity in the association between ADs and smoking behavior. After controlling for demographics, depression and drug abuse/dependence, associations between increased odds of lifetime and 12-month daily smoking were observed with PTSD (Lifetime: OR 1.58 (1.21 to 2.06); 12-month: OR 1.46 (1.08 to 1.97)), 12-month daily smoking with PD (OR 1.42 (1.04 to 1.94)), and Lifetime daily smoking with GAD (OR 1.23 (1.05 to 1.61)).

In a nationally representative sample of the New Zealand population, individuals with ADs (grouped) had a smoking prevalence of 30.4% (27.7 to 33.0), and consumed approximately 16% of all cigarettes in New Zealand [7]. In Australia, data from the nationally representative National Survey of Mental Health and Wellbeing 2007 reported rates of current and daily smoking in those with individual ADs (range for current smoker: 33% to 45%; range for daily smoker: 27% to 42%) well above the rates in respondents not reporting a mental disorder (current: 13.6%; daily: 10.8%) [6].

Cougle *et al. *[44] demonstrated increased odds of ND in patients with SP (OR 1.31 (1.01 to 1.71)), GAD (OR 1.59 (1.21 to 1.98)) and PTSD (OR 1.47 (1.01 to 2.16)) when adjusting for demographics, depression and drug abuse/dependence. Utilizing the NESARC for adults aged 18 to 25 years [46], the odds of 12-month ND were significantly increased for respondents with lifetime specific phobia (OR 1.8 (1.16 to 2.88)) after controlling for other psychiatric disorders, smoking and demographic characteristics.

### Anxiety disorder by smoking or nicotine dependence status

Utilizing the German Transitions in Alcohol Consumption and Smoking study, Schumann *et al. *[64] calculated unadjusted odds ratios for individual ADs based on smoking and ND status. When comparing ND ever smokers (respondents with ND who had smoked at least one cigarette daily for ≥ 4 weeks at some point in their life) to non-ND ever smokers, increased odds were found for PD (OR 2.92 (1.78 to 2.73)), SP (OR 3.07 (1.70 to 5.57)), specific phobia (OR 2.09 (1.63 to 2.68)), GAD (OR 4.26 (1.85 to 9.84)) and PTSD (OR 2.08 (1.13 to 3.83)). Grant *et al. *[52] used data from NESARC to compare the odds of ADs on ND status. Unadjusted odds ratios with individual ADs as dependent variables were greater across all assessed ADs. Point estimates for odds ratios ranged from 2.6 for SP to 4.6 for PD with agoraphobia for respondents with versus without ND. Data from the UK National Households Survey [49] demonstrated increased rates of GAD (4.1% vs 2.4%), specific phobia (1.5% vs 0.8%) and PD (1.5% vs 0.5%) in respondents with ND versus those without ND. Degenhardt *et al. *[45] utilized the 1997 National Survey of Mental Health and Wellbeing in Australia to demonstrate increased odds of ADs (grouped) in current smokers versus never smokers (OR 1.50 (1.21 to 1.87)) when adjusted for demographic status, other drug use and neuroticism.

## Discussion

Our systematic review of the literature revealed a total of 17 studies that provided prospective or quasiprospective information regarding the relationship between ADs, ND and cigarette smoking. Of these studies 14 provided prospective or quasiprospective information on the role of ADs on the risk of smoking or ND, and 7 studies provided the role of daily smoking or ND on onset of ADs. A further 31 studies reported only cross-sectional relationships between smoking, ND and ADs. The association between smoking, ND and ADs could be explained by three non-mutually exclusive relationships; smoking and ND leads to increased ADs, the reverse association, or a shared vulnerability model where a factor or group of factors increase smoking, ND and AD expression.

### Are anxiety disorders a risk factor for smoking onset?

A relatively common hypothesis links increased anxiety with smoking onset and increased smoking behaviors. Multiple studies conducted in both general and clinical populations have demonstrated increased rates of smoking amongst individuals with all AD subtypes [68-70], although exceptions exist [71]. The results of these prospective, quasiprospective and cross-sectional studies are inconsistent, and importantly, many of these studies have failed to take into account the important role of comorbid substance use disorders. In a study of patients with ADs where those with alcohol and substance use disorders were excluded, rates of smoking were lower than the control population [72], indicating comorbid illness is important in understanding this association.

This review found a limited and heterogeneous literature evaluating the prospective effect of ADs on smoking onset in random population samples. The available data varied significantly on population characteristics (whole population vs specific age range), length of prospective follow-up (1 year to 10 years), diagnostic tool utilized and smoking definition. On balance, the 10-year follow-up of the National Comorbidity Survey [34] represents the likely best evidence available to date. From this analysis, baseline PD, SP, GAD and specific phobia, but not PTSD or agoraphobia, were associated with increased odds of commencing daily smoking. There are however important considerations in interpreting this study. First, the diagnostic classification system utilized in the first NCS (DSM-III-R) was updated in the NCS2 (DSM-IV). Although there is a large concordance between these diagnostic classifications across most ADs, some significant differences exist, particularly in the criteria of PTSD, which may have altered relative prevalence rates [73]. Second, and most importantly, the presented analyses did not control for the presence of other psychiatric disorders or for alcohol or drug abuse/dependence. As noted by Cougle *et al. *[44], ADs are often comorbid disorders, particularly with MDD and substance abuse disorders. In other presented prospective studies, univariate relationships between PD and smoking onset failed to sustain significance when comorbid MDD was included in the analytic models [19], and the presence of a comorbid substance use disorder reversed the direction of effect of baseline AD from protective to causative in the NESARC cohort [33]. The NCS follow-up revealed larger increased odds of daily smoking onset in respondents with alcohol or other substance abuse/dependence (OR point estimates between 2.6 and 4.2). As such, it is probable that some of the association between ADs and smoking is due to effects of comorbid substance abuse disorders.

Considering that most of the other available prospective studies failed to find significant associations between ADs and subsequent onset of smoking, it is currently difficult to draw definitive conclusions regarding AD as a risk factor for smoking. One potential factor that should be taken into account is the populations considered in the available literature. Population-based studies suggest that the average age individuals first use cigarettes has been declining, and now ranges between 12 years and 16 years [74]. In addition, smokers who commence earlier are more likely to persist than those that commence at a later age [75]. Given that most of the included studies assessed adult populations, it is possible many participants with ADs were already smoking and hence new onset smoking was relatively rare in assessed populations. In contrast to cross-sectional analyses, very limited prospective information is available on varying characteristics of smoking behavior (for example, persistence) and ADs in the available prospective studies.

Some studies have used a quasiprospective design, pairing cross-sectional data with retrospective self-report data, to assess the impact of ADs on smoking status. Such strategies are limited by recall bias, and any conclusions drawn must consider this. Analysis of the NCS by Breslau *et al. *[35] found increased odds of daily smoking in those with a variety of pre-existing or current ADs. However, these analyses did not control for the effects of comorbid substance use disorders or other psychiatric disorders and, additionally, were limited to onset of daily smoking as opposed to smoking more generally. When considering the effects of pre-existing ADs on smoking persistence, no significant associations were found.

A number of theories are proposed to explain why ADs may lead to increased rates of smoking. Theoretical explanations are informed by both psychological (for example, conditioning theory, cognitive theory, anxiety sensitivity [76]) and biological (for example, nicotine effects and withdrawal) factors. One explanatory model relates to the use of cigarettes as an anxiolytic agent (that is, self-treatment). Evidence supports that nicotine exposure does produce a subjective calming effect, although this is coupled with an increase in objective measures of physiological arousal [77]. Tachyphylaxis and homeostatic adjustment however implies that acute and chronic effects of cigarettes, as with any agents that induce tolerance and dependence, may differ substantially. Treatment of this extensive literature is beyond this review, although we refer readers to a number of excellent comprehensive reviews on this subject [78-81], which include identification of the methodological challenges in exploring the effect of anxiety traits and states on smoking behaviors.

### Are anxiety disorders a risk factor for later nicotine dependence?

Inconsistent although clearer associations between baseline ADs and subsequent ND were found in the literature. In the 10-year follow-up of the NCS [34], having baseline PTSD, agoraphobia and specific phobia were associated with increased odds of onset of ND amongst already smokers, and PTSD, SP and specific phobia were associated with increased odds for ND amongst the whole population. The clear association between baseline PTSD and ND was replicated by Breslau *et al. *[21] in the Detroit Epidemiologic Study, while Isensee *et al. *[27] found baseline PD to be associated with increased risk for subsequent ND. Given anxiety disorders are associated with negative affect [82], and negative affect is linked to increased smoking motivation [83], it is possible that negative affect may underpin an increased likelihood to developing ND in those with ADs. Further evidence demonstrating an effect of duration of anxiety disorder diagnosis and later ND severity would be useful.

### Is smoking or nicotine dependence a risk factor for onset of anxiety disorders?

A limited number of prospective studies are available assessing smoking or ND as risk factors for incident ADs in population-based epidemiological studies. The available literature supports ND as increasing the odds of later PTSD, and smoking as increasing the odds for some (PD and GAD), but not all, ADs and interpretation of some analyses are complicated by non-inclusion of potential confounders. For example, the prospective odds of PD onset were increased in the Oregon Adolescent Depression Project, the New York Adolescent Cohort and the EDSP in individuals with a history of prior smoking versus non-smokers. This result remained significant when controlling for other ADs in the Oregon Adolescent Depression Study, but failed to maintain significance when comorbid psychiatric disorders were controlled for in the EDSP study. However, smoking as a risk factor for PD is supported by the Detroit Epidemiologic Study, which found increased risk of PD onset in prior daily smokers, with a stronger association in prior daily smokers who continued to smoke. These analyses controlled for MDD, but not other psychiatric or substance use disorders. The use of quasiprospective methodology on data from the NCS allowed further exploration of this relationship. These data suggested that daily smoking was a risk factor for increased odds of PD and agoraphobia, with analyses controlling for other psychiatric disorders including substance use disorders [38]. A demonstrated dose-dependent relationship would support a causal association between cigarette smoking and subsequent onset of ADs. Breslau demonstrated that increased standardized pack years of smoking were associated with increased odds of GAD, but decreased odds of PD [38] in dose dependent fashion.

The interaction between panic, PD and cigarette smoking has received more investigation in epidemiological and laboratory-based studies than other ADs, and the evidence was recently reviewed [84]. The literature has demonstrated that earlier age of smoking initiation is associated with increased risk of PD [85], and smokers demonstrated earlier PD onset in comparison to non-smokers [86]. Given the somatic nature of panic attack symptomatology, it is possible that smoking may be more likely to produce panic-type AD symptoms secondary to the physical effects of smoking (for example, respiratory and autonomic disturbance). Panic is influenced by respiratory sensitivity, and nicotine alters the sensitivity set point of cholinergic respiratory neurons [87]. From their review of the literature, Cosci *et al. *[84] nominated three potential hypotheses underpinning the association between cigarette smoking and panic. The first is a 'moderational model', whereby neuroticism moderates the panic/smoking association. The second is a 'pathoplastic model', where smoking influences PD expression by 'exacerbating affective disturbances and negative health process' [88], including a combination of direct negative health effects (for example, induction of chronic obstructive pulmonary disease (COPD)), acute physiological effects (for example, increased noradrenaline and autonomic sensations) and negative self-perception of health status [89]. The third is the 'false suffocation alarm' theory, proposed by Klein [90], who suggested that smoking may induce hypersensitivity to suffocation signals and increase the risk of experiencing panic. The suffocation false alarm may potentially be mediated by episodic dysfunction in regulation of endogenous opioid function [91].

A number of studies have assessed the interaction of smoking and PTSD. A population-based follow-up investigation of individuals affected by a fireworks factory explosion in The Netherlands found that those who were smokers at time of traumatic incident had an increased chance of experiencing severe anxiety symptoms (OR 2.32 (1.19 to 4.53)) and disaster related PTSD (OR 2.64 (1.05 to 6.62)) at 4-year follow-up, controlling for baseline symptoms, demographic characteristics and life events [92]. This supports the results of Koenen *et al. *[36], who demonstrated that the presence of ND increased the risk of developing PTSD in trauma exposed men (HR 1.98 (1.61 to 2.42)) and that shared genetic effects explained 63% of the ND-PTSD association. A number of neurobiological (for example, alterations to the hypothalamic-pituitary-adrenal axis, sympathetic nervous system hyperactivity alteration to neurotransmitter system functioning) and psychological factors may contribute to a PTSD, ND and cigarette smoking association (see Fu *et al. *[93] for review). ND may increase ADs by exacerbating anxiety responses [94], or by facilitating other psychiatric states (for example, depression) that may predispose individuals to develop anxiety. In addition, the number of ND symptoms and nicotine withdrawal symptoms has been robustly correlated in a dose-dependent fashion with increasing anxiety disorder diagnosis [56]. In reference to other ADs, supportive evidence was found for smoking increasing the odds of developing GAD, while no evidence was found to support smoking as a risk factor for onset of SP, although some evidence supports the reverse association in female ND smokers [28].

In assessing smoking or ND as a risk factor for ADs, one factor that must be considered is that ADs have been estimated to exhibit a median age of onset of only 6 years [95]. Given the early age of AD development, from early childhood through adolescence, exposure to environmental cigarette smoke may influence AD development. Exposure to second-hand smoke has been demonstrated to be positively associated with symptoms of GAD, but not PD in children and adolescents age 8 years to 15 years [96]. Additionally, early onset smoking (10 to 15 years) has been associated with an earlier onset of ADs when compared to late onset smoking (> 15 years) [97]. It is possible that exposure to cigarette smoke in critical periods of brain development may be one factor that predisposes individuals to subsequent develop ADs. A number of biological pathways may underpin this effect, including alteration in neurotransmitter function, and induction of oxidative and nitrosative stress that may overwhelm intrinsic defenses inhibiting normal neuronal cell functions, including neuroplasticity and neurogenesis.

### Shared vulnerability between smoking, ND and ADs

A number of shared vulnerability factors contribute to cigarette smoking behavior and ADs, including early childhood experiences and environment [83,98,99], problems with impulsivity [100] and distress tolerance and neurotic personality traits [101,102]. Indeed, it is possible that these shared vulnerability factors are further underpinned by other factors (for example, genetics, fetal insults), and ADs and cigarette smoke could alter their expression (for example, cigarette smoking may reinforce poor distress tolerance). For a more detailed discussion of the role and interaction of shared vulnerability factors see the review by Morissette *et al. *[79].

### Limitations and directions for future research

It is important to consider the limitations of this study design in interpreting the results. This review was designed deliberately to only consider random, large general population samples where validated structured diagnostic tools were utilized to make diagnoses. Effort was made to identify all studies meeting inclusion criteria, although it is possible some important sources of information were not identified, particularly from non-English language journals. The inclusion criteria were designed to allow for selection of only the best quality evidence not restricted by certain biases (for example, selection bias, recall bias) although as consequence of this a number of identified studies involving samples drawn from clinic populations [72,103-105] and studies which utilized anxiety symptom scores [8] or non-validated diagnostic tools (for example, self-report) as end points were excluded from final analysis. The heterogeneous nature of included studies, particularly in regards to population demographics; smoking definition; diagnostic criteria utilized; diagnostic group included; and comorbid factors included in analysis also complicates interpretation. A number of future studies would assist in clarifying issues from this review. First, the continuation of current prospective cohorts over longer periods and the recruitment of new cohorts, incorporating and measuring changes to smoking behavior over time, would assist in clarifying the relationship between smoking and ADs. Similarly, given the early onset nature of many ADs, maternal and birth cohorts assessing these issues would assist greatly in deepening our understanding. Exploration of the effects of quitting on anxiety and depression would also clarify associations. In addition, further exploration of potential biological mechanisms underpinning associated effects of smoking and ADs would help to elucidate shared vulnerabilities underpinning both ADs and smoking.

## Conclusions

A large literature has demonstrated that individuals with ADs have higher rates of smoking and ND than those without ADs, although most extant information is drawn from cross-sectional studies. In terms of prospective studies, there exists a limited heterogeneous literature examining the relationship between cigarette smoking, ND and ADs in population-based epidemiological studies utilizing structured diagnostic tools linked to ICD or DSM criteria. The available prospective evidence provides support for smoking and ND as being risk factors for the onset of PD and GAD, although varied results are found across studies. In addition, some ADs have been associated with subsequent onset of smoking and ND, although many studies failed to include comorbid substance use disorders and other psychiatric disorders in analyses.

## Competing interests

SM declares no conflicts of interest in relation to this article. FNJ has received grant/research support from the Brain and Behaviour Research Institute, the National Health and Medical Research Council, Australian Rotary Health, the Geelong Medical Research Foundation, the Ian Potter Foundation, Eli Lilly and The University of Melbourne and has been a paid speaker for Sanofi-Synthelabo, Janssen Cilag and Eli Lilly. She is supported by an NHMRC Training Fellowship (#628912). JAP has received speaker fees from Amgen, Eli Lilly and Sanofi-Aventis and funding from the Geelong Region Medical Research Foundation, Barwon Health, Perpetual Trustees, the Dairy Research and Development Corporation, The University of Melbourne, the Ronald Geoffrey Arnott Foundation, ANZ Charitable Trust, the American Society for Bone and Mineral Research, Amgen (Europe) GmBH and the NHMRC. MB has received Grant/Research Support from the NIH, Cooperative Research Centre, Simons Autism Foundation, Cancer Council of Victoria, Stanley Medical Research Foundation, MBF, NHMRC, Beyond Blue, Geelong Medical Research Foundation, Bristol Myers Squibb, Eli Lilly, Glaxo SmithKline, Organon, Novartis, Mayne Pharma and Servier, has been a speaker for Astra Zeneca, Bristol Myers Squibb, Eli Lilly, Glaxo SmithKline, Janssen Cilag, Lundbeck, Merck, Pfizer, Sanofi Synthelabo, Servier, Solvayand Wyeth, and served as a consultant to Astra Zeneca, Bristol Myers Squibb, Eli Lilly, Glaxo SmithKline, Janssen Cilag, Lundbeck and Servier.

## Authors' contributions

SM conceived of the study, developed the methods, conducted the literature search and led the development of the final manuscript. FNJ, JAP and MB contributed to the study design and data analysis, and provided intellectual content to the final manuscript. All authors read and approved the final manuscript.

## Pre-publication history

The pre-publication history for this paper can be accessed here:

http://www.biomedcentral.com/1741-7015/10/123/prepub

## Supplementary Material

Additional file 1 **Table S1**. Prospective longitudinal studies investigating influence of anxiety disorders on subsequent risk of smoking and nicotine dependence (ND). Click here for file

Additional file 2 **Table S2**. Prospective longitudinal studies investigating influence of smoking and nicotine dependence (ND) on subsequent risk of anxiety disorders. Click here for file

Additional file 3 **Table S3**. Quasiprospective studies investigating influence of anxiety disorders on subsequent risk of smoking and nicotine dependence (ND). Click here for file

Additional file 4 **Table S4**. Quasiprospective studies investigating influence of smoking and nicotine dependence (ND) on subsequent risk of anxiety disorders. Click here for file

Additional file 5 **Table S5**. Cross-sectional studies investigating associations between anxiety disorders, nicotine dependence (ND) and smoking. Click here for file
